# Genomic characterisation of *Leptospira inadai* serogroup Lyme isolated from captured rat in Brazil and comparative analysis with human reference strain

**DOI:** 10.1590/0074-02760170444

**Published:** 2018-03-12

**Authors:** Luisa Z Moreno, Fabiana Miraglia, Ana P Loureiro, Frederico S Kremer, Marcus R Eslabao, Odir A Dellagostin, Walter Lilenbaum, Silvio A Vasconcellos, Marcos B Heinemann, Andrea M Moreno

**Affiliations:** 1 Universidade de São Paulo, Faculdade de Medicina Veterinária e Zootecnia, Laboratório de Epidemiologia Molecular e Resistência a Antimicrobianos, São Paulo, SP, Brasil; 2 Universidade Federal Fluminense, Departamento de Microbiologia e Parasitologia, Laboratório de Bacteriologia Veterinária, Niterói, RJ, Brasil; 3 Universidade Federal de Pelotas, Centro de Desenvolvimento Tecnológico, Pelotas, RS, Brasil

**Keywords:** Leptospira inadai, serogroup Lyme, comparative genomic analysis

## Abstract

*Leptospira inadai* is classified as a species of the *Leptospira* intermediate group that has been poorly studied due to its apparent insignificance to human and animal health. Nevertheless, over the last two decades the species has been described in human cases in India and in carrier animals in Ecuador. Here, we present the first identification and genomic characterisation of *L. inadai* serogroup Lyme isolated from captured rodent in Brazil. Even though the M34/99 strain was not pathogenic for hamsters, it was able to establish renal colonisation. The M34/99 strain presented high similarity with *L. inadai* serogroup Lyme human reference indicating that animal strain could also infect humans, although it does not represent high risk of severe disease. An extrachromosomal sequence was also identified in M34/99 strain and presented high identity with previously described *L. inadai* phage LinZ_10, suggesting that phage-like extrachromosomal sequence may be another feature of this understudied species.

Leptospirosis is a worldwide zoonosis, with higher incidence in tropical climates, caused by bacteria of the *Leptospira* genus (Levett 2001, Evangelista and Coburn 2010). To date, the genus comprises 10 pathogenic species, seven non-pathogenic species, and an intermediate group containing five species (Bourhy et al. 2014). The intermediate clade includes *Leptospira inadai*, *L. broomii*, *L. fainei*, *L. wolffii* and *L. licerasiae*, of which *L. wolffii* and *L. licerasiae* have been associated with mild leptospirosis (Matthias et al. 2008, Slack et al. 2008, Zakeri et al. 2010), while *L. broomii* and *L. fainei* have been reported from severe cases of the disease (Arzouni et al. 2002, Levett et al. 2006).

*L. inadai* was first isolated from a skin biopsy of a patient with Lyme disease, in 1981 (Schmid et al. 1986). Even though the strain was classified as pathogenic to laboratory animals, it was also considered only a concurrent infection in the patient. The isolated strain (strain 10) is considered the species type strain that was only formally described by Yasuda et al. (1987). Over the last two decades, *L. inadai* was only reported in two fatal human cases in India (Gangadhar et al. 2008) and in carrier animals in Ecuador (Chiriboga et al. 2015). Here, we present the identification, genomic characterisation and comparative analysis of *L. inadai* serogroup Lyme isolated from captured rodent in Brazil.

The M34/99 strain was isolated in 1999 from the kidney of a captured urban brown rat (*Rattus norvegicus*) in São Paulo city. The strain was included in the *Leptospira* collection of the Laboratory of Bacterial Zoonosis - University of São Paulo, with inconclusive results for identification, and stored in Fletcher’s medium (DIFCO/USA), enriched with 15% rabbit serum and maintained in EMJH broth (DIFCO/USA) at 30ºC.

Serogrouping was performed at the Laboratory of Veterinary Bacteriology -Fluminense Federal University. The isolate was subjected to microscopic agglutination test (MAT) using a panel of polyclonal rabbit antisera of 32 reference serovars representing the 24 known serogroups (provided by Royal Tropical Institute - KIT, Amsterdam). The M34/99 strain presented high agglutination rates with serogroup Lyme antisera (25,600).

Virulence assessment was performed with five Golden Syrian hamsters (*Mesocricetus auratus*); animals were infected with M34/99 strain (10^8^ leptospires) through intraperitoneal route. The animal experiment was conducted with the approval of the Ethics Committee from the School of Veterinary Medicine and Animal Science - University of São Paulo (2244/2011). Clinical symptoms were checked daily for 21 consecutive days. All animals survived the challenge and were euthanised and necropsied to assess renal infection. Kidney samples were aseptically collected, homogenised in 50 mL of Sorensen saline and 100 µL aliquots of 10^-1^ to 10^-3^ dilutions were inoculated in Fletcher medium and incubated at 30ºC for six weeks. Even though the M34/99 strain was not pathogenic for hamsters, it could establish renal colonisation as the hamsters kidney samples enabled recovery of the M34/99 strain.

For species identification and complete genetic characterisation, the M34/99 strain was submitted to whole-genome sequencing. Genomic DNA was purified with illustra^™^ bacteria genomicPrep Mini Spin Kit (GE Healthcare do Brasil Ltda, São Paulo, Brazil) and used for paired-end library preparation with Nextera^™^ DNA Sample Prep Kit (Illumina^®^) and sequencing through Illumina^®^ Miseq platform. The de novo assembly was performed with Geneious R10 (Biomatters Ltd, Auckland, New Zealand) and resulted in 59 scaffolds with a N_50_ of 163,176 bp. Automatic genome annotation was performed with NCBI Prokaryotic Genome Annotation Pipeline (Tatusova et al. 2016).

The species identification was confirmed by phylogenetic analysis of 16S rRNA and *rpoB* sequences performed with Mega 5.10 software (Tamura et al. 2011) with maximum-likelihood method and Tamura-Nei model, using reference sequences from GenBank. Strain identification was also confirmed by pairwise comparison of average nucleotide identity (ANIb) trough JSpeciesWS (Richter et al. 2016). The phylogenetic analysis resulted in the *L. inadai* identification for both genes (

Supplementary data). The ANIb results (Table) also confirmed the species identification (> 99% of nucleotide identity with *L. inadai* serovar Lyme strain 10 genome) and differentiation from the remaining species of the *Leptospira* intermediate group.

**TABLE:** Pairwise comparison of average nucleotide identity (ANIb) for M34/99 strain and the *Leptospira* intermediate group

Strain	Nucleotide identity (%)					
*L. inadai* M34/99						
*L. inadai* 10	99.97					
*L. broomii* 5399	89.88	89.89				
*L. fainei* BUT6	85.69	85.70	86.55			
*L. wolffii* Khorat-H2	70.46	70.46	70.32	70.19		
*L. licerasiae* VAR010	69.94	69.94	69.89	69.91	73.23	

The obtained scaffolds were ordered according to *L. inadai* serovar Lyme strain 10 (NZ_AHMM00000000.2) using Mauve Multiple Genome Aligner (Darling et al. 2010). Due to the unavailability of a complete *L. inadai* reference genome, the chromosomes of the studied genome were not individualised. The M34/99 draft genome (MCRM00000000.2) comprises ~4.56 Mb and only one scaffold (scaffold 22, ~87 kb) did not align with the reference strain 10. BLASTn against non-redundant NCBI database was applied for the extrachromosomal sequence and resulted in high identity (>99%) with *L. inadai* strain 10 phage LinZ_10 (KF114880).

The *L. inadai* drafts genomes (strains 10 and M34/99) were compared to the closely related species *L. broomii* serovar Hurstbridge (NZ_AHMO00000000) and *L. fainei* serovar Hurstbridge (NZ_AKWZ00000000) through Artemis Comparison Tool (ACT) (Carver et al. 2005) and BLAST Ring Image Generator (BRIG) (Alikhan et al. 2011). *L. inadai c*hromosomal content presented high synteny and 99% identity at DNA level among studied strains (Fig. 1). It is also worth noting a few deletion regions in *L. broomii* and *L. fainei* compared to *L. inadai*; although the species present some similarity of content (> 80% genetic similarity) (Fig. 1A), the sequences also present arrangement variation (blocks of inverted sequences among the genomes) (Fig. 1B).

**Fig. 1:**
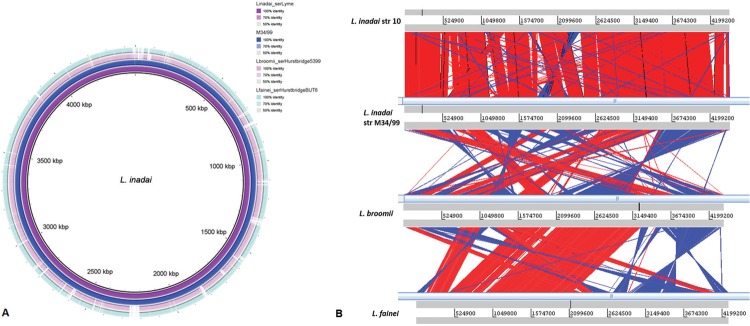
: whole genome comparative analysis of *Leptospira inadai* serogroup Lyme strains 10 and M34/99 (NZ_AHMM00000000.2 and MCRM00000000.2) with *L. broomii* serovar Hurstbridge (NZ_AHMO00000000) and *L. fainei* serovar Hurstbridge (NZ_AKWZ00000000). (A) BRIG plot displaying genomic similarity. (B) Artemis Comparison Tool (ACT) synteny visualisation.

With regard to the virulence genes, M34/99 strain presents only a few genes encoding lipoproteins (*lipA*) and flagellar proteins (*fliG/F*), as expected. In addition, resistance genes were identified, including antimicrobial resistance genes (*tetA*), efflux pumps (*mdtC*, *norM*), and heavy metal and antiseptic resistance genes (*merR*, *sugE*, *qacA*). Fouts et al. (2016) had already demonstrated that *L. inadai* serovar Lyme strain 10 lacked some of *Leptospira* major virulence factors, such as LipL32 and LipL41, which are markers of pathogenic *Leptospira* and, therefore, could explain *L. inadai* isolation from asymptomatic hosts. Considering that fatal *L. inadai* infection has only been reported in India (Gangadhar et al. 2008), these strains require further study to ensure the species and genetic mechanisms of virulence identification.

Regarding the *L. inadai* extrachromosomal content, M34/99 phage-like sequence was deposited in GenBank as Lin_34 under the accession number KX656785. The sequence, of 87,3 kb, only differs from the reference *L. inadai* serovar Lyme strain 10 LinZ_10 bacteriophage by ~2,3 kb due to the absence of IS66 family proteins in strain M34/99. Both sequences are mostly composed of hypothetical proteins and although the gene content is very similar, Mauve and ACT analyses demonstrate that there are blocks of inverted sequences (Fig. 2) that appear to be related to the presence of insertion sequences in LinZ_10 bacteriophage.

**Fig. 2:**
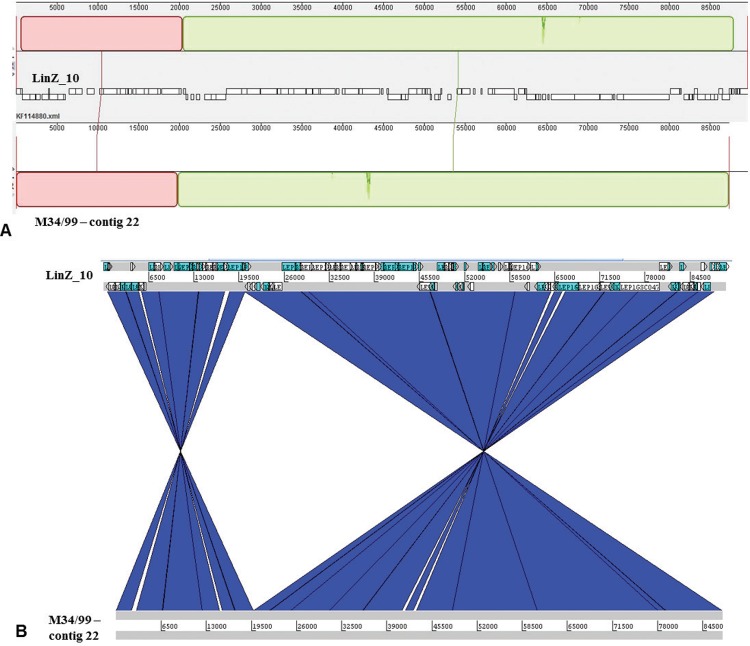
: comparative analysis of *Leptospira inadai* phage-like extrachromosomal sequences from Brazilian M34/99 strain and strain 10 phage LinZ_10 (KF114880). (A) Mauve alignment blocks. (B) Artemis Comparison Tool (ACT) synteny visualisation of extrachromosomal element (blue blocks link regions that are homologous but inverted with respect to each other).

This is the first report of *L. inadai* in Brazil and the second of this species in South America; it is an important epidemiological data of the *Leptospira* species circulating in the country. *L. inadai* has been described as highly adapted to rodents in India (Gangadhar et al. 2000) and in Ecuador the species has also been recovered from dogs, pigs, bovines and rats (Chiriboga et al. 2015). The identification of a Brazilian *L. inadai* strain isolated from urban rat corroborates that the species is adapted to rodents as a reservoir and it also suggests that *L. inadai* presents a wider dissemination that probably goes unnoticed because it is not part of the routine antigen battery of diagnostics laboratories.

The high similarity of *L. inadai* serogroup Lyme rodent stain with the genome of the human strain indicates that the species could able to infect humans even though it does not represent a high risk of severe disease. Considering the geographical and chronological distance and the high genomic similarity observed between strains, this may suggest a possibility of a similar lineage of *L. inadai* serogroup Lyme in the Americas. The phage identification in the Brazilian *L. inadai* serogroup Lyme strain, as well as in strain 10 original description (LinZ_10), also suggests that phage-like extrachromosomal sequence may be another common feature of this understudied species.

## References

[B1] Alikhan NF, Petty NK, Ben Zakour NL, Beatson SA (2011). BLAST Ring Image Generator (BRIG): simple prokaryote genome comparisons. BMC Genomics.

[B2] Arzouni JP, Parola P, La Scola B, Postic D, Brouqui P, Raoult D (2002). Human infection caused by Leptospira fainei. Emerg Infect Dis.

[B3] Bourhy P, Collet L, Brisse S, Picardeau M (2014). Leptospira mayottensis sp. nov., a pathogenic species of the genus Leptospira isolated from humans. Int J Syst Evol Microbiol.

[B4] Carver TJ, Rutherford KM, Berriman M, Rajandream MA, Barrell BG, Parkhill J (2005). ACT: the Artemis Comparison Tool. Bioinformatics.

[B5] Chiriboga J, Barragan V, Arroyo G, Sosa A, Birdsell DN, España K (2015). High prevalence of intermediate Leptospira spp. DNA in febrile humans from urban and rural Ecuador. Emerg Infect Dis.

[B6] Darling AE, Mau B, Perna NT (2010). progressiveMauve: multiple genome alignment with gene gain, loss and rearrangement. PLoS ONE.

[B7] Evangelista KV, Coburn J (2010). Leptospira as an emerging pathogen: a review of this biology, pathogenesis and host immune responses. Fut Microbiol.

[B8] Fouts DE, Matthias MA, Adhikarla H, Adler B, Amorim-Santos L, Berg DE (2016). What makes a bacterial species pathogenic? Comparative genomic analysis of the genus Leptospira. PLoS Negl Trop Dis.

[B9] Gangadhar NL, Prabhudas K, Bhushan S, Sulthana M, Barbuddhe SB, Rehaman H (2008). Leptospira infection in animals and humans: a potential public health risk in India. Rev Sci Tech.

[B10] Gangadhar NL, Rajasekhar M, Smythe LD, Norris MA, Symonds ML, Dohnt MF (2000). Reservoir hosts of Leptospira inadai in India. Rev Sci Tech.

[B11] Levett P, Morey R, Galloway R, Steigerwalt A (2006). Leptospira broomii sp. nov., isolated from humans with leptospirosis. Int J Syst Evol Microbiol.

[B12] Levett PN (2001). Leptospirosis. Clin Microbiol Rev.

[B13] Matthias MA, Ricaldi JN, Cespedes M, Diaz MM, Galloway RL, Saito M (2008). Human leptospirosis caused by a new, antigenically unique Leptospira associated with a Rattus species reservoir in the Peruvian Amazon. PLoS Negl Trop Dis.

[B14] Richter M, Rosselló-Móra R, Glöckner FO, Peplies J (2016). JSpeciesWS: a web server for prokaryotic species circumscription based on pairwise genome comparison. Bioinformatics.

[B15] Schmid GP, Steere AC, Kornblatt AN, Kaufmann AF, Moss CW, Johnson RC (1986). Newly recognized Leptospira species (‘‘Leptospira inadai’’ serovar lyme) isolated from human skin. J Clin Microbiol.

[B16] Slack AT, Kalambaheti T, Symonds ML, Dohnt MF, Galloway RL, Steigerwalt AG (2008). Leptospira wolffii sp. nov., isolated from a human with suspected leptospirosis in Thailand. Int J Syst Evol Microbiol.

[B17] Tamura K, Peterson D, Peterson N, Stecher G, Nei M, Kumar S (2011). MEGA5: molecular evolutionary genetics analysis using maximum likelihood, evolutionary distance, and maximum parsimony method. Mol Biol Evol.

[B18] Tatusova T, DiCuccio M, Badretdin A, Chetvernin V, Nawrocki EP, Zaslavsky L (2016). NCBI prokaryotic genome annotation pipeline. Nucleic Acids Res.

[B19] Yasuda PH, Steigerwalt AG, Sulzer KR, Kaufmann AF, Rogers F, Brenner DJ (1987). Deoxyribonucleic acid relatedness between serogroups and serovars in the family Leptospiraceae with proposals for seven new Leptospira species. Int J Syst Bacteriol.

[B20] Zakeri S, Khorami N, Ganji ZF, Sepahian N, Malmasi AA, Gouya MM (2010). Leptospira wolffii, a potential new pathogenic Leptospira species detected in human, sheep and dog. Infect Genet Evol.

