# Supplementary material for: Genomic characterisation of Leptospira inadai serogroup Lyme isolated from captured rat in Brazil and comparative analysis with human reference strain
Source: Mem Inst Oswaldo Cruz. 2018 Mar 12;113(5):e170444. doi: 10.1590/0074-02760170444 (PMC5851024; doi:10.1590/0074-02760170444)
Supplement: Supplementary file 1 [file 0074-0276-mioc-113-5-e170444-suppl01.pdf]

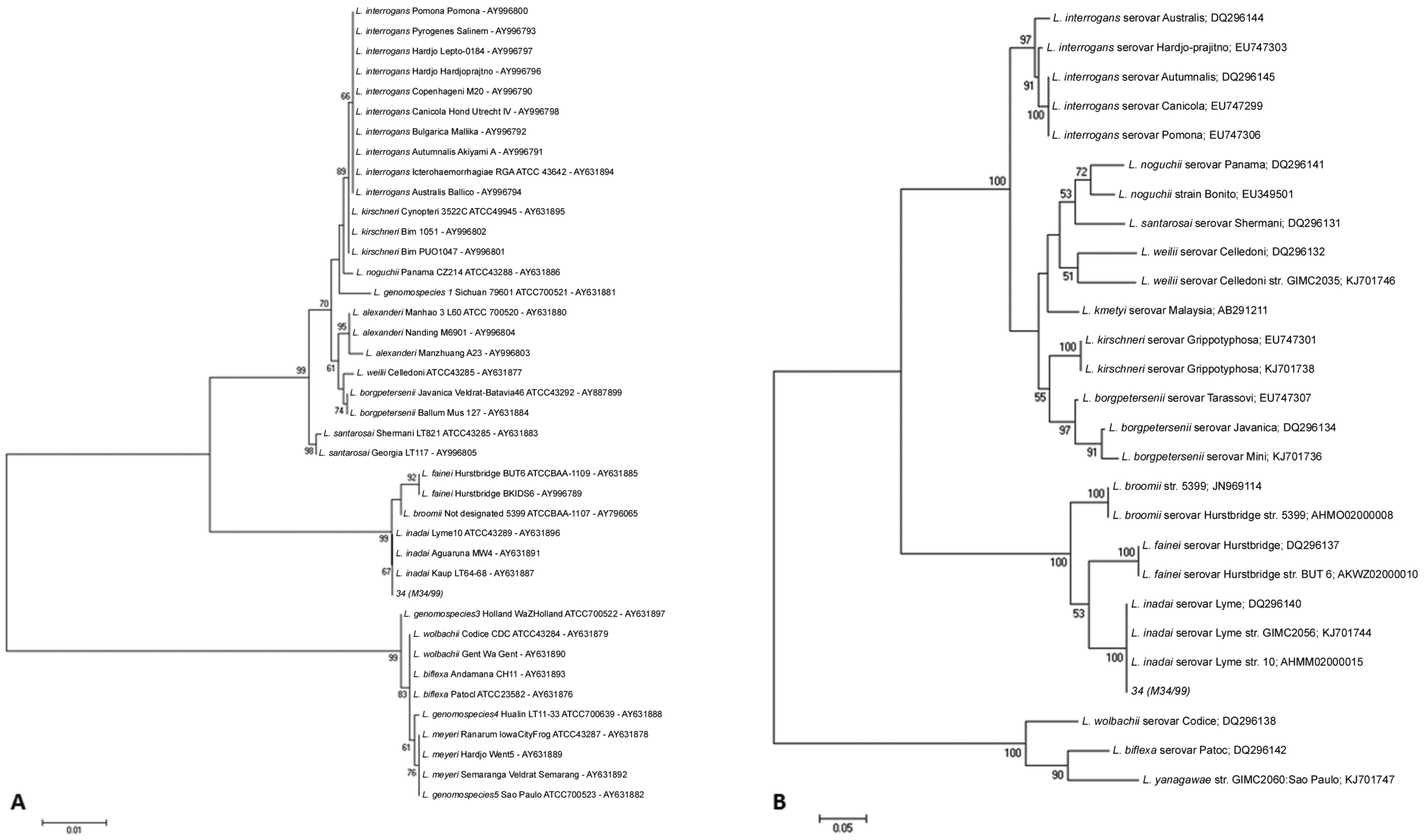

Maximum-likelihood tree based on the 16S rRNA (A) and *rpoB* (B) nucleotide sequences. The bootstrap values presented at corresponding branches were evaluated using 500 replicates. The Brazilian *Leptospira inadai* serogroup Lyme M34/99 strain is highlighted in bold type clustered together with *L. inadai* reference sequences from GenBank database.

cc BY
